# Effect of the pipeline embolization device placement on branching vessels in anterior circulation: a systematic review and meta-analysis

**DOI:** 10.1007/s00701-024-05895-5

**Published:** 2024-01-11

**Authors:** Yiming He, Tao Sun, Mengtao Han, Donghai Wang

**Affiliations:** 1 Department of Neurosurgery and Shandong Key Laboratory of Brain Function Remodeling, Qilu Hospital of Shandong University, Jinan, 250000 Shandong China; 2 Department of Neurosurgery, Qilu Hospital of Shandong University Dezhou Hospital (Dezhou, China), Cheeloo Hospital of Shandong University, Jinan, 250000 Shandong China

**Keywords:** Pipeline embolization device, Cerebral aneurysm, Side branch, Systematic review, Meta-analysis

## Abstract

**Background and purpose:**

Pipeline embolization device (PED) is widely used in intracranial aneurysms, and the scope of applications for the PED, which is frequently used to treat cerebral aneurysms, is also growing. It has some effect on branching vessels as a result of its inherent properties. The effects of PED on the complications rate and branching vessels blockage have not yet been thoroughly investigated.

**Objective:**

We conducted a systematic review searching reports from multiple databases on PED use for intracranial aneurysms, and analyzed the influence of PED on the occlusion rate of different branching vessels, and the influence of the amount of PED on the occlusion rate of branching vessels by meta-analysis.

**Methods:**

We searched the literature using PUBMED, Web of Science, and OVID databases until August 2023. Inclusion criteria were that the study used only PED, included at least 10 patients, and recorded branching vessels occlusion rates, mortality, and neurological complications.

**Results:**

Nine studies were analyzed consisting of 706 patients with 986 side branches. The results of the meta-analysis showed that application of more than one PED did not significantly elevate the rate of branching vessels occlusion compared to application of one PED (OR = 0.70; 95% CI: 0.34 to 1.43; *P* = 0.33). In the comparison of branching vessels occlusion rates in the anterior circulation, the anterior cerebral artery (ACA) had a significantly higher occlusion rate compared to the ophthalmic artery (OphA) (OR = 6.54; 95% CI: 3.05 to 14.01; *P* < 0.01), ACA also had a higher occlusion rate compared to the anterior choroidal artery (AchA) (OR = 15.44; 95% CI: 4.11 to 57.94 *P* < 0.01), ACA versus posterior communicating artery (PcomA) occlusion rate difference was not statistically significant (OR = 2.58; 95% CI: 0.63 to 12.82; *P* = 0.17), OphA versus AchA occlusion rate difference was not statistically significant (OR = 2.56; 95% CI: 0.89 to 7.38; *P* = 0.08), and the occlusion rate was significantly higher for PcomA compared to AchA (OR = 7.22; 95% CI: 2.49 to 20.95; *P* < 0.01) and lower for OphA compared to PcomA (OR = 0.33; 95% CI: 0.19 to 0.55; *P* < 0.01).

**Conclusion:**

The meta-analysis shows that use of multiple PEDs did not significantly increase the occlusion rate of branching vessels, and the larger the diameter of branching vessels covered by PED, the higher the occlusion rate of branching vessels. However, the incidence of complications is low after branching vessels occlusion in anterior circulation, which is related to the collateral circulation compensation of the branching vessels.

## Introduction

Intracranial aneurysms (IAs) are common vascular lesions with a prevalence of approximately 1–6% in the general population [3, 16, 18]. While IAs have historically been treated by surgical clipping or bypass, both of which are invasive procedures requiring craniotomies, endovascular intervention have since been developed as less invasive treatment alternatives. Placement of detachable coils, stents, and flow diverter devices (FDD) can be performed using endovascular approaches [3, 5]. For small cerebral aneurysms, stent-assisted coil embolization has a high rate of aneurysm occlusion, but for large and wide-necked aneurysms, the coil cannot fill the aneurysm lumen, resulting in a low rate of aneurysm occlusion, so this type of aneurysm requires flow diverter devices [10, 14, 22].

The current widely used flow diverter device is the pipeline embolization device (PED, Medtronic, Irvine, CA). PED are used for larger wide-necked carotid aneurysms of the internal carotid artery, and because of their low porosity and high pore density, they can effectively promote the occlusion of aneurysms, but it can also easily lead to the occlusion of parent arteries and branching vessels [8]. Perforator artery occlusion after PED treatment is a major concern, as it may lead to corresponding neurological complications.

Through the search, no systematic review and meta-analysis of branching vessels after PED treatment were found. We aim to conduct a systematic review and meta-analysis to determine the occlusion of branching vessels and its related complications after PED treatment.

## Methods

This systematic review and meta-analysis was conducted and reported in accordance with the Meta-Analysis of Observational Studies in Epidemiology (MOOSE) guidelines and the PRISMA statement. The review was registered at the International Prospective Register of Systematic Reviews (PROSPERO; https://www.crd.york.ac.uk/PROSPERO/) as registration number CRD42022349449.

### Search strategy

We searched the articles using PUBMED, Web of Science and OVID databases until August 2023. The search terms were as follows: “intracranial aneurysm,” “brain aneurysm,” “cerebral aneurysm,” “PED,” “pipeline embolization device,” “pipeline,” “side branches,” “side branch,” “collateral circulation,” “branching vessel,” “branching vessels,” “occlusion,” “(vessel) patency,” and each search term was connected with Boolean operators OR and AND. In addition, the references of the obtained articles were retrieved to expand the study.

### Selection criteria

The selection criteria were as follows: (a) patients treated with PED; (b) number of patients was at least 10; (c) rate of branching vessels occlusion was documented in the study; (d) written in English.

The criteria for exclusion were as follows: (a) non-PED-treated patients, or the PED treatment modalities that accounted for only a portion of the study; (b) studies with less than 10 cases; (c) case report, review, and experimental animal studies; (d) written in languages other than English.

### Quality assessment

All included case-control studies and cohort studies were quality assessed by two authors (Yiming He and Tao sun) via the Newcastle-Ottawa Quality Assessment Scale (NOS) (Table 1), and studies with a score of 6 or higher were included in the meta-analysis, and any questions regarding disagreement on quality assessment were addressed by a third author (Mengtao Han) to resolve.

**Table 1:** Detailed quality assessment of cohort studies

Items of NOS	Studies						
	G. Gascou	T.R. Miller	Xinzhi Wu	Scott	Aditya	Leonardo	Simon Chun-Ho Yu
Selection							
Is the case definition adequate?	*	*	*	*	*	*	*
Representativeness of the cases	*	*	*	*	*	*	*
Selection of Controls	*	*	*	*	*	*	*
Definition of Controls							
Comparability							
Comparability of cases and controls on the bias of the design or analysis		**	**	*	*	**	**
Exposure							
Ascertainment of exposure	*	*	*	*	*	*	*
Same method of ascertainment for cases and controls	*	*	*	*	*	*	*
Non-Response rate	*	*	*	*	*	*	*
Total	6	8	8	7	7	8	8

A study can be awarded a maximum of one star for each numbered item thin the Selection and Outcome categories. A maximum of two stars can be given for Comparability. Studies’ rates ≥ 6 are eligible. *NOS*, Newcastle-Ottawa Scale

### Data extraction

Two authors (Yiming He and Tao Sun) read through eligible studies from which the following data were extracted: author, country, year, study design, sample size, number of side branches covered by PEDs, mean diameter of PEDs applied to occluded or patent side branches, associated neurological complication rates, side branches occlusion rates with one PED or multiple PEDs applied, occlusion rate of different side branches.

### Endpoints

Primary outcome of this meta-analysis was the incidence of side branches occlusion treated with PED. According to different studies, we usually adopt the occlusion rate of side branches at the last follow-up of angiography. For the study, which included the incidence of stenosis and occlusion, only the occlusion rate is used. Other outcomes include the occlusion rate of side branches caused by different numbers of PED, the incidence of neurological complications related to side branches occlusion.

### Statistical analysis

Our study consisted of case-control study (6 cases) and a cohort study (1 case), so odds ratio (OR) and 95% confidence intervals were used to assess the difference in occlusion rates between the two groups. We used the Review.

Manager software (RevMan version 5.3, the Nordic Cochrane Center, the Cochrane Collaboration, 2014) software to perform the meta-analysis. We assessed heterogeneity using the I^2^ statistic and Cochrane *Q* test. *I*^2^ values of 25%, 50%, and 75% represent low, medium, and large differences, respectively. *p* values less than 0.05 indicate statistically significant differences. *I*^2^ < 50% used a fixed-effects model, and *I*^2^ > 50% used a random-effects model.

## Result

### Literature review

**Fig. 1:**
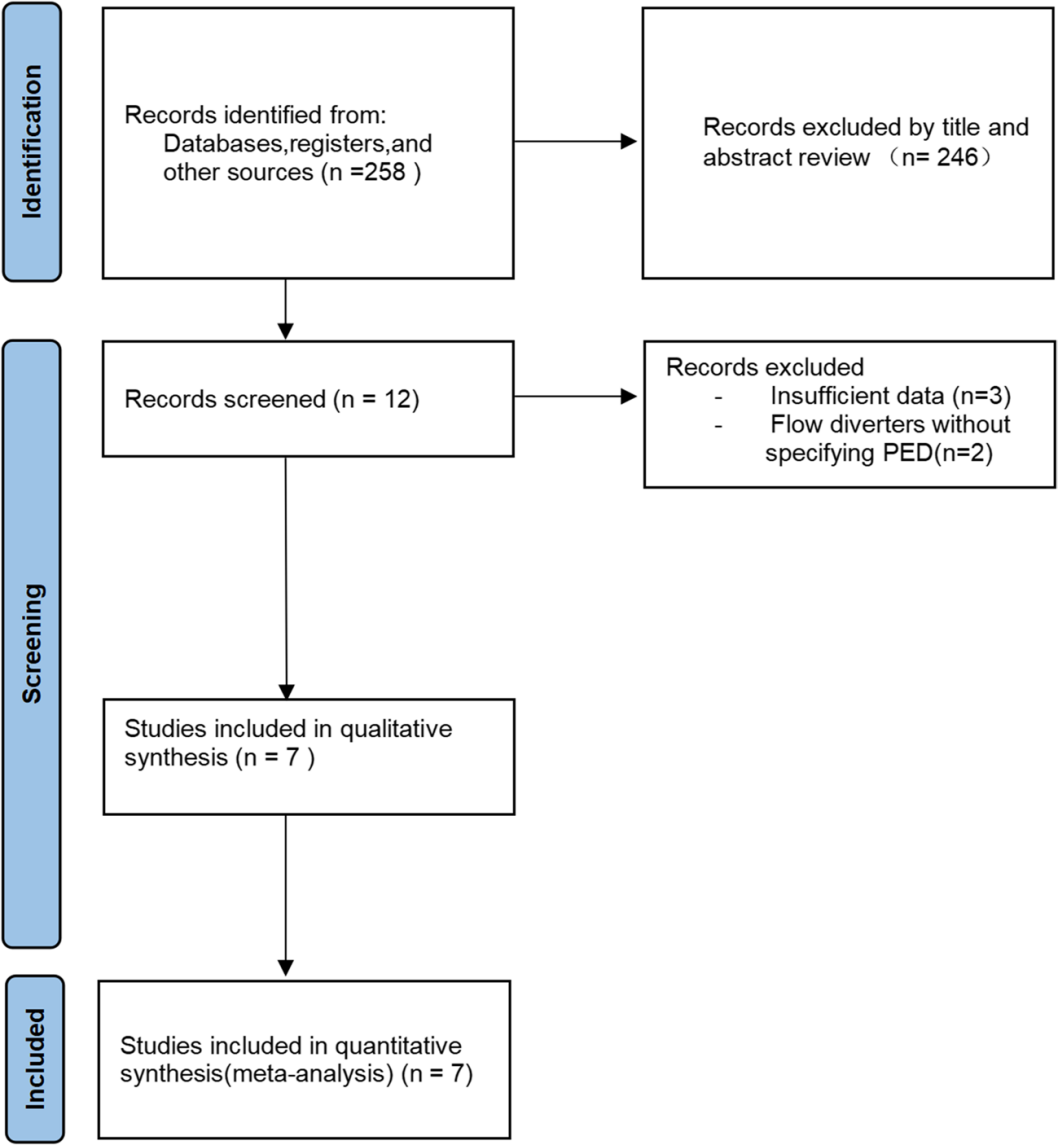
PRISMA flow diagram of literature retrieval. PRISMA, preferred reporting items for systematic reviews and meta-analyses

Two hundred fifty-eight publications were retrieved through Pubmed, Web of Science, and OVID. 246 publications were excluded by reading the titles and abstracts of the literature, and after reading the full text, 5 publications were again excluded due to lack of relevant outcome data in some of them. Seven literatures were finally included in the study, containing six case–control studies and one cohort study (Fig. 1). All studies included a total of 680 patients and 960 side branches.

### Characteristics of the included studies

All included articles were in English, six studies were retrospective and one was prospective, with publication dates from 2012 to 2019 and sample sizes of 59 to 143. In our meta-analysis, 680 patients were treated with PED in all studies, covering 960 collateral branches. Most of the articles only studied the branching vessels of the anterior circulation, and some of the articles recorded the branching vessels of the posterior circulation (Table 2).

**Table 2:** Baseline characteristics of the included studies

Study(Year)	Study Type	Number of Patients	Number of Aneurysms	Number of Branches covered	Branch occlusion	PED diameter for BO	PED diameter without BO
G.Gascou (2015)	RCS	59	66	68	2(3)	NR	NR
T.R.Miller (2018)	RCS	137	178	217	29(13.4)	4 ± 0.5	3.5 ± 0.5
Xinzhi Wu (2019)	RCS	126	NR	173	26(15)	4.2 ± 0.5	3.9 ± 0.4
Aditya (2014)	RCS	49	68	74	3(4)	NR	NR
Leonardo (2016)	RCS	82	82	127	13(10.2)	NR	NR
Scott (2018)	RCS	84	89	127	17(13.3)	NR	NR
Simon Chun-Ho Yu (2012)	PCS	143	178	174	NR	NR	NR
Study(Year)	One PED Occlusion	Multi-PED Occlusion	OphA Occlusion	AhcA Occlusion	PcomA Occlusion	ACA Occlusion	Complications
G.Gascou (2015)	1(1.8)	1(7.7)	2(8)	0	0	0	0
T.R.Miller (2018)	28(14)	1(5.5)	8(6)	1(3.2)	13(33.3)	4(36.3)	0
Xinzhi Wu (2019)	22(13.4)	2(22.2)	12(11)	0	4(16.7)	7(63.6)	0
Aditya (2014)	NR	NR	2(4)	0	1(7.1)	NR	NR
Leonardo (2016)	7(15.2)	10(27.7)	8(10.5)	0	3(10.7)	2(100)	0
Scott (2018)	NR	NR	7(10)	1(4)	7(28)	0	NR
Simon Chun-Ho Yu (2012)	2(1.3)	0	0	0	2(5.4)	0	NR

*RCS*, retrospective cohort study; *PCS*, prospective cohort study; *NR*, not reported; *BO*, branch occlusion; *PED*, pipeline embolization; *OA*, ophthalmic artery; *AchA*, anterior choroidal artery; *PcomA*, posterior communicating artery; *ACA*, anterior cerebral artery

### Study outcomes

**Fig. 2:**
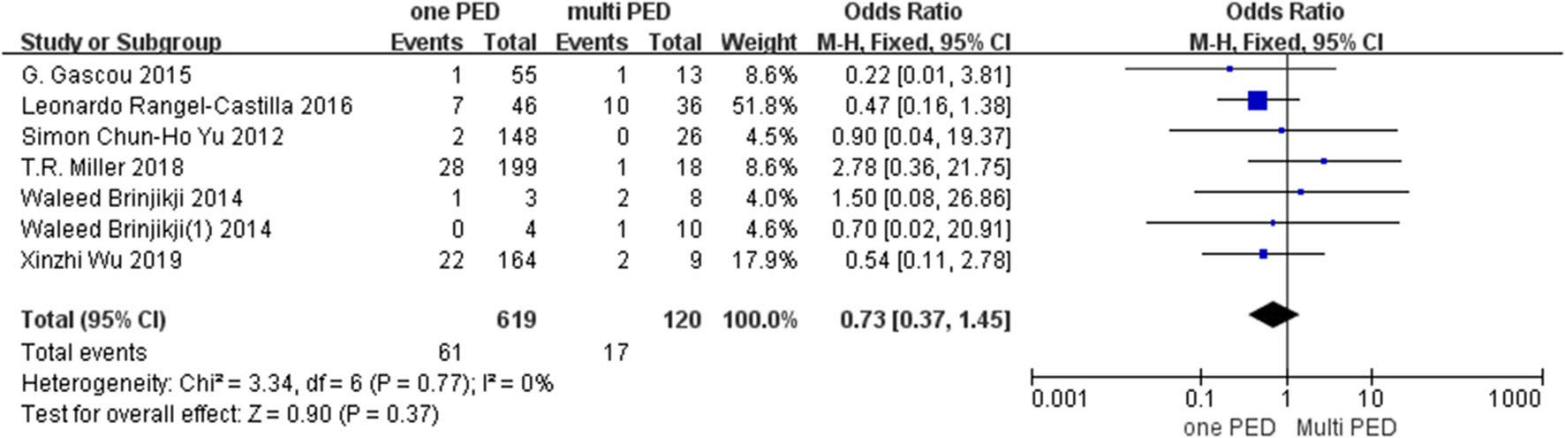
Forest plot showing the effects of single PED and multiple PED on side branches occlusion

**Fig. 3:**
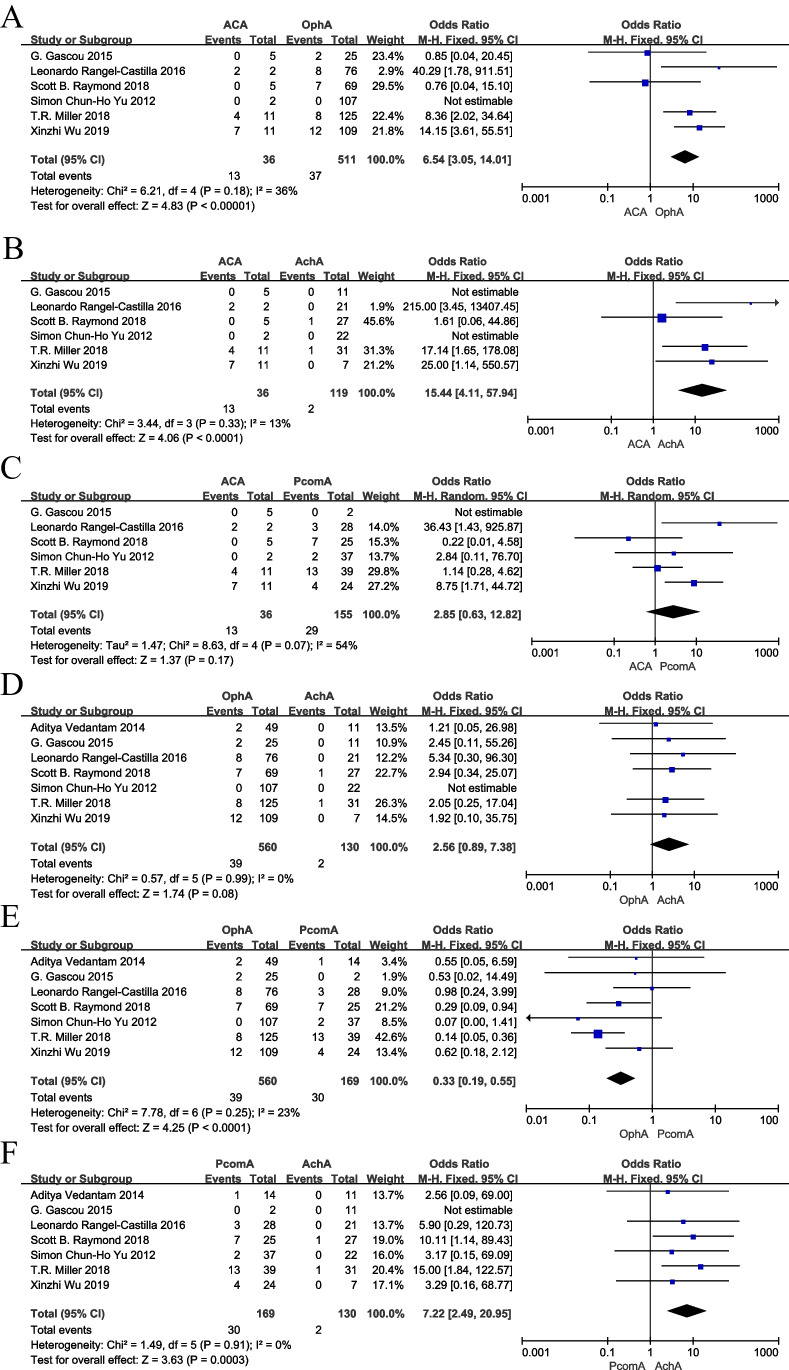
Forest plot showing the comparison of occlusion rates of different side branches. **A** ACA versus OphA; **B** ACA versus AchA; **C** ACA versus PcomA; **D** OphA versus AchA; **E** OphA versus PcomA; **F** PcomA versus AchA

In the included articles, the angiographic follow-up results of most of the literatures showed that the postoperative occlusion rate of ACA (0–100%, mean occlusion rate 33.3%) was significantly higher than that of other side branches. Even though some articles showed that the occlusion rate of ACA was lower, it also had a higher stenosis rate compared with other side branches, while the postoperative occlusion rate of AchA (0–4%, mean occlusion rate 1.2%) was significantly lower than that of other side branches. The results of the meta-analysis showed that use of more than one PED did not significantly elevate the rate of branching vessels occlusion compared to application of one PED (OR = 0.70; 95% CI: 0.34 to 1.43; *P* = 0.33) (Fig. 2). In the comparison of branching vessels rates in the anterior circulation, the anterior cerebral artery (ACA) had a significantly higher occlusion rate compared to the ophthalmic artery (OphA) (OR = 6.54; 95% CI: 3.05 to 14.01; *P* < 0.01) (Fig. 3), ACA also had a higher occlusion rate compared to the anterior choroidal artery (AchA) (OR = 15.44; 95% CI: 4.11 to 57.94 *P* < 0.01), ACA versus posterior communicating artery (PcomA) occlusion rate difference was not statistically significant (OR = 2.58; 95% CI: 0.63 to 12.82; *P* = 0.17), OphA versus AchA occlusion rate difference was not statistically significant (OR = 2.56; 95% CI: 0.89 to 7.38; *P* = 0.08), and the occlusion rate was significantly higher for PcomA compared to AchA (OR = 7.22; 95% CI: 2.49 to 20.95; *P* < 0.01) and lower for OphA compared to PcomA (OR = 0.33; 95% CI: 0.19 to 0.55; *P* < 0.01). In the comparison of different groups, most of the groups were less heterogeneous and we used a fixed-effects model, but the heterogeneity between the ACA and PcomA groups was higher (I2 > 50%, *p* = 0.07), so we used a random-effects model for this group.

## Discussion

Through meta-analysis, we found that in the anterior circulation, ACA had a higher occlusion rate after PED, followed by OphA and PcomA, and AchA had the lowest occlusion rate. There was no significant difference in branching vessels occlusion rate between single PED and multiple PED. It has been pointed out that branching vessels occlusion is related to collateral blood supply [11, 12]. ACA, OphA, and PcomA are supplied by anterior communicating artery, external carotid artery and posterior cerebral artery respectively. Proximal vascular occlusion is easy to be caused by pressure gradient, while AchA has no collateral blood supply, so the occlusion rate is low. Some studies have shown that there is a correlation between the branching vessels occlusion rate and the diameter of branching vessels, the larger the branching vessels diameter, the higher the postoperative occlusion rate. Hu et al. used CFD to study patient-specific AICAs covered by virtual implanted PEDs, the reduction rate of blood flow was 3.6 ± 1.9%. They found that larger branch diameters were associated with higher blood flow reduction rates, which indirectly explains why ACA has a high occlusion rate [6].

The study of Leonardo found that the increase of the number of PED would increase the occlusion rate of lateral branches [12], but other studies showed that the occlusion rate of lateral branches had nothing to do with the number of PED. Through the meta-analysis, we found that there was no significant correlation between the number of PED and the occlusion rate of lateral branches. The studies of T.R. Miler and Xinzhi Wu show that the diameter of PED is related to branching vessels occlusion [9, 19]. The smaller the diameter of PED, the higher the occlusion rate of branching vessels, which may be because the smaller the diameter is, the higher the pore density is. In the study of Chao Xu [20], the ratio of PED diameter to ipsilateral internal carotid artery was negatively correlated with branching vessels occlusion rate (univariate analysis 0.012, multivariate analysis 0.013), but the standard deviation and the number of PED in different groups were not given in the three studies. Moreover, the number of articles is too little. Therefore, meta-analysis cannot be performed.

The related complications caused by branching vessels occlusion are low in the literature, even most of the literature reported that after branching vessels occlusion, the related complications are zero [4, 13, 17, 21]. In the study of Chao Xu et al., one patient developed A1 occlusion, and the mRS score of the last follow-up was two, with A1 occlusion-related complications [20]. In another study, one patient had an acute occlusion of the ophthalmic artery caused by PED covering the ophthalmic artery, resulting in small visual field deficit after surgery, while there were no related complications in other patients with delayed occlusion of the ophthalmic artery [15]. In the study of Rishi et al., the left posterior inferior cerebellar artery (PICA) occlusion was caused by the implantation of PED for posterior circulation aneurysm, and the patient developed dysarthric speech after awakening under general anesthesia [7]. From the above results, we found that acute branching vessel occlusion caused by PED implantation is more likely to have related complications, while patients with delayed occlusion often has no complications, because patients with delayed occlusion have a longer occlusion time, and the collateral circulation of branching vessels compensate for blood supply, thus making patients less prone to related complications. However, acute occlusion results in sudden decrease of blood flow in branching vessels, which is more prone to occlusion-related complications. Many studies have found that compared with the lateral branches with lower occlusion rate, the related neurological complications of the lateral branches with high occlusion rate are lower. For the lateral branches with higher occlusion rate, it may be related to the blood supply of branches [2, 12, 19]. Moreover, there is a higher complication rate after occlusion of perforator arteries in the posterior circulation than in the anterior circulation, possibly because the posterior circulation arteries are generally thinner and lack branches to compensate [1].

This study has several limitations. First, less articles and data may affect the meta-analysis results of some branches. Second, only two articles reported the effect of PED diameter on branching vessels occlusion, so we did not analyze the results of PED diameter. Third, because the articles searched are all about branching vessels occlusion of anterior circulation aneurysms, there are few articles about branching vessels occlusion of posterior circulation aneurysms. Therefore, meta-analysis cannot be carried out, so this paper only discusses the problem of branching vessels occlusion of anterior circulation aneurysms.

## Conclusion

When PED is applied to intracranial aneurysms, after covering the branching vessels, it will affect the branching vessels in varying degrees, ACA had a higher rate of occlusion in the anterior circulation, followed by PcomA and OphA. AchA had the lowest occlusion rate, indicating that the larger the diameter of branching vessels covered by PED, the higher the occlusion rate of branching vessels. However, the larger the diameter of branching vessel after occlusion, the lower the incidence of related complications. There is no significant correlation between the amount of PED and the occlusion rate of branching vessels. At present, there is a lack of study on the effect of PED diameter on branching vessels occlusion and the effect of PED on posterior circulation branching vessels. In the future, more studies are needed to determine whether the diameter of PED has an effect on branching vessels occlusion, and more studies are also needed to determine the difference in occlusion rate of each branching vessels of posterior circulation after PED placement and the difference of occlusion rate between anterior and posterior circulation branching vessels.

## Data Availability

Not applicable.

## References

[1] Brinjikji W et al (2013) Endovascular treatment of intracranial aneurysms with flow diverters: a meta-analysis. Stroke 44(2):442–447 23321438 10.1161/STROKEAHA.112.678151

[2] Brinjikji W et al (2014) Patency of the posterior communicating artery after flow diversion treatment of internal carotid artery aneurysms. Clin Neurol Neurosurg 120:84–88 24731582 10.1016/j.clineuro.2014.02.018

[3] Chan DY et al (2016) Screening for intracranial aneurysms? Prevalence of unruptured intracranial aneurysms in Hong Kong Chinese. J Neurosurg 124(5):1245–1249 26473778 10.3171/2015.4.JNS142938

[4] Gascou G et al (2015) Extra-aneurysmal flow modification following pipeline embolization device implantation: focus on regional branches, perforators, and the parent vessel. AJNR Am J Neuroradiol 36(4):725–731 25523592 10.3174/ajnr.A4191 PMC7964318

[5] Guglielmi G et al (1991) Electrothrombosis of saccular aneurysms via endovascular approach. Part 2: Preliminary clinical experience. J Neurosurg 75(1):8–14 2045924 10.3171/jns.1991.75.1.0008

[6] Hu P et al (2015) Blood flow reduction of covered small side branches after flow diverter treatment: a computational fluid hemodynamic quantitative analysis. J Biomech 48(6):895–898 25748223 10.1016/j.jbiomech.2015.02.015

[7] Lall RR et al (2014) Acute branch occlusion after Pipeline embolization of intracranial aneurysms. J Clin Neurosci 21(4):668–672 24156905 10.1016/j.jocn.2013.07.011

[8] Liou TM, Li YC (2008) Effects of stent porosity on hemodynamics in a sidewall aneurysm model. J Biomech 41(6):1174–1183 18377914 10.1016/j.jbiomech.2008.01.025

[9] Miller TR et al (2018) Pipeline diameter significantly impacts the long-term fate of jailed side branches during treatment of intracranial aneurysms. AJNR Am J Neuroradiol 39(12):2270–2277 30385475 10.3174/ajnr.A5863 PMC7655415

[10] Pai AM, Kameda-Smith M, van Adel B (2018) A review of recent advances in endovascular therapy for intracranial aneurysms. Crit Rev Biomed Eng 46(4):369–397 30806250 10.1615/CritRevBiomedEng.2018027003

[11] Puffer RC et al (2012) Patency of the ophthalmic artery after flow diversion treatment of paraclinoid aneurysms. J Neurosurg 116(4):892–896 22224787 10.3171/2011.11.JNS111612

[12] Rangel-Castilla L et al (2017) Patency of anterior circulation branch vessels after Pipeline embolization: longer-term results from 82 aneurysm cases. J Neurosurg 126(4):1064–1069 27285547 10.3171/2016.4.JNS16147

[13] Raymond SB et al (2018) The role of collateral circulation in branch vessel occlusion after flow diversion. World Neurosurg 10.1016/j.wneu.2018.12.064 30593960

[14] Shin DS et al (2020) The evolution of flow-diverting stents for cerebral aneurysms; historical review, modern application, complications, and future direction. J Korean Neurosurg Soc 63(2):137–152 32120455 10.3340/jkns.2020.0034 PMC7054118

[15] Szikora I et al (2010) Treatment of intracranial aneurysms by functional reconstruction of the parent artery: the Budapest experience with the pipeline embolization device. AJNR Am J Neuroradiol 31(6):1139–1147 20150304 10.3174/ajnr.A2023 PMC7963954

[16] Thompson BG et al (2015) Guidelines for the management of patients with unruptured intracranial aneurysms: a guideline for healthcare professionals from the American Heart Association/American stroke association. Stroke 46(8):2368–2400 26089327 10.1161/STR.0000000000000070

[17] Vedantam A et al (2015) Incidence and clinical implications of carotid branch occlusion following treatment of internal carotid artery aneurysms with the pipeline embolization device. Neurosurgery 76(2):173–8; discussion 178. 10.1227/NEU.0000000000000595 10.1227/NEU.0000000000000595 25549190

[18] Vlak MH et al (2011) Prevalence of unruptured intracranial aneurysms, with emphasis on sex, age, comorbidity, country, and time period: a systematic review and meta-analysis. Lancet Neurol 10(7):626–636 21641282 10.1016/S1474-4422(11)70109-0

[19] Wu X et al (2019) Patency of branch vessels after pipeline embolization: comparison of various branches. Front Neurol 10:838 31440201 10.3389/fneur.2019.00838 PMC6694210

[20] Xu C et al (2021) Safety evaluation and flow modification in the anterior cerebral artery after pipeline embolization device deployment across the internal carotid artery terminus. Biomed Res Int 2021:6657595 34471639 10.1155/2021/6657595 PMC8405287

[21] Yu SC et al (2012) Intracranial aneurysms: midterm outcome of pipeline embolization device–a prospective study in 143 patients with 178 aneurysms. Radiology 265(3):893–901 22996749 10.1148/radiol.12120422

[22] Zhao J et al (2018) Current treatment strategies for intracranial aneurysms: an overview. Angiology 69(1):17–30 28355880 10.1177/0003319717700503 PMC5724574

